# Two successive bidirectional leaders propagated in triggered lightning channel

**DOI:** 10.1038/s41598-022-12522-9

**Published:** 2022-06-02

**Authors:** Junlin Wang, Quanxin Li, Jianguo Wang, Li Cai, Rui Su, Mi Zhou, Yadong Fan

**Affiliations:** School of Electrical Engineering and Automation, Wuhan University, Wuhan, China

**Keywords:** Atmospheric science, Applied physics, Plasma physics

## Abstract

A stepped leader propagated along the previous return-stroke channel in triggered lightning. After the stepped leader decayed, the first bidirectional leader went through the process of initiation, propagation and dissipation. Then the second bidirectional leader initiated at the termination of the decayed first bidirectional leader and propagated toward the ground, generating the fourth return-stroke. The observations were synchronously performed through a high-speed camera and electromagnetic field measurements. The first bidirectional leader was characterized by similar average upward and downward velocities of 0.76 × 10^6^ m/s and 0.67 × 10^6^ m/s. The velocity of the upward positive leader of the first bidirectional leader was noticeably fluctuated, ranging 0.39 × 10^6^–1.78 × 10^6^ m/s. The second bidirectional leader was characterized by a sustainable propagating upward end with an average velocity of 1.82 × 10^6^ m/s. The velocity fluctuation trend of the upward end depends on the neutralization amount of the residual negative charge and the positive charge in UPL.

## Introduction

Kasemir first proposed and developed the bidirectional leader concept, in which model, two leader ends, carrying charge of opposite polarity, propagate simultaneously in opposite directions^1^. Various observations had verified this concept^2–6^. For instance, two polarity leaders are usually initiated from the opposite end of the triggering wire and propagate bidirectionally in altitude-triggered lightning^3,7^. And the recoil leader usually propagated bidirectionally within the remnant positive leader channel^2,4,8,9^. In addition, the bidirectional leaders developing in the virgin air or a decayed channel during the late stage of a cloud discharge were observed by Montanyà et al.^5^ and Tran and Rakov^10^.

However, there are relatively few bidirectional leaders, which initiated at the termination of a decayed dart/dart-stepped leader, developed within the preconditioned main return-stroke channel^6,11^. Jiang et al.^11^ observed bidirectional propagation of a dart leader developing through the preconditioned channel. They reported that the dart leader initially propagated downward and terminated at an altitude of about 2.2 km, and then a bidirectional leader initiated at the termination of the decayed dart leader. Qie et al.^6^ observed a phenomenon that dart or dart-stepped leaders propagated along the preexisting channel and terminated at an altitude of about 300 m to 500 m, then a bidirectional leader initiated at the termination of the former decayed leader. The common point was that the one bidirectional leader initiated at the termination of the former decayed dart/dart-stepped leader, and finally reached the ground, generating a return-stroke. And the whole process took place in the preexisting channel.

In this paper, two successively initiated bidirectional leaders propagating in one preexisting channel after the stepped leader decayed were observed in triggered lightning, which was significantly different from the one bidirectional leader after decayed dart/dart-stepped leader in Jiang et al.^11^ and Qie et al.^6^. The first bidirectional leader (BL-1) occurred following the termination of the decayed stepped leader, and the second bidirectional leader (BL-2) occurred following the termination of the decayed BL-1. Optical characteristics and correlated electromagnetic field features are analyzed. To date, such two successively initiated bidirectional leaders’ development in one leader/return-stroke sequence is reported with fine synchronous data for the first time.

### Analysis and results

**Figure 1:**
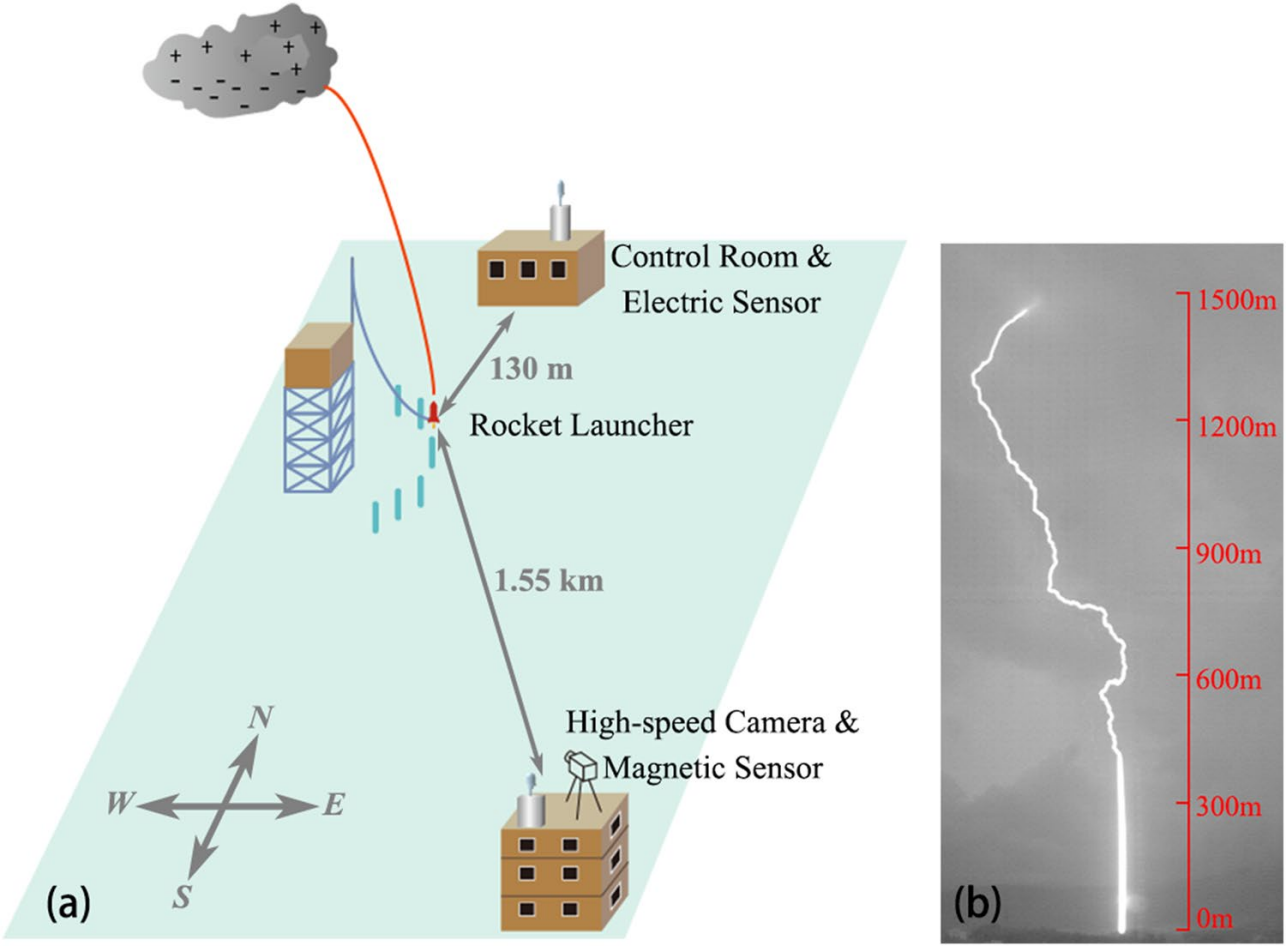
Sketch of the equipment including the high-speed camera, magnetic sensor, rocket launcher and electric sensor. Artificially Triggered lightning Experiment had been carried out in the summer of 2019 in Guangzhou Field Experiment Site for Lightning Research and Testing (GFESLRT), and the flash (F201906301713) analyzed in the letter was triggered at 17:13:13 on 30 June, 2019. (**a**) The layout of the equipment including the high-speed camera, magnetic sensor and electric sensor. (**b**) The image of the lightning channel and the corresponding height.

There were altogether nine return-strokes (RS) in F201906301713. A feebly luminous channel of the third return-stroke path was found to be propagated along by a stepped leader in triggered lightning. Two successively initiated bidirectional leaders occurred after the stepped leader decayed, then generating the fourth return-stroke. All RSs processes were conventional sequence of dart leader/return-stroke^12^, excluding the case of two successive bidirectional leaders/return-stroke (RS4) in the present study. The experiment equipment was shown in Fig. 1.

**Figure 2:**
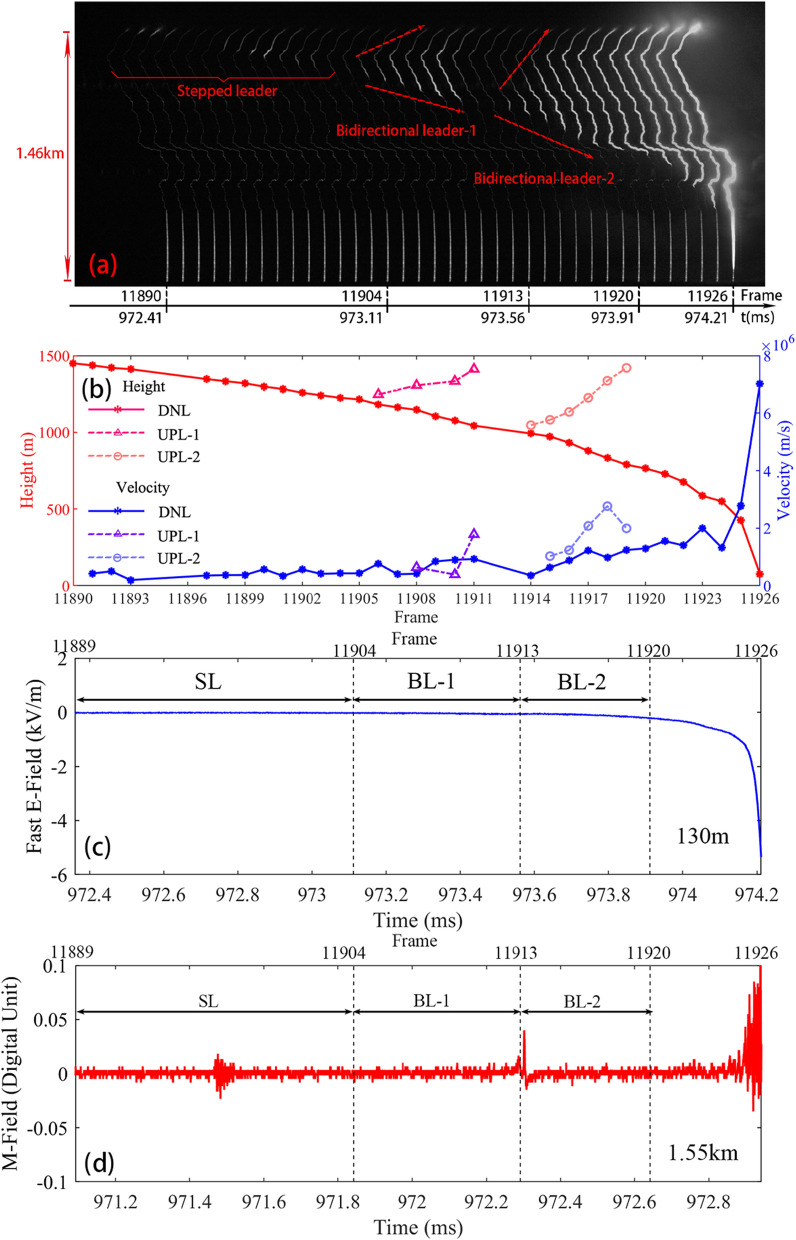
Overview of the leader process before the RS4. (**a**) Image with arranged consecutive high-speed video frames revealing the development of stepped leader and bidirectional leaders. The time interval between two consecutive frames was 50 μs (20,000 fps); (**b**) variations of the height and the 2-D estimated velocity of both upward positive leader (UPL) and downward negative leader (DNL); (**c**) electric field change 130 m away from the rocket launcher; (**d**) Magnetic field 1.55 km away from the rocket launcher; There was a delay of 1.27 ms between the waveforms measured at 130 m and 1.55 km respectively. The number of frames of several high-speed video corresponding to the waveform was marked on the top of the graph.

Figure 2 shows the overview of the leader process before the RS4. Figure 2a shows the developing process of the bidirectional leaders following by a return-stroke (RS4). The background noise was removed and the image contrast was enhanced for a better presentation. The lightning channel over 1.46 km was blocked by clouds. Before the stepped leader development, the whole channel was blurry with low-level luminosity through the channel remnant caused by the continuous current. The stepped leader started propagating downward from Frame 11,890. The frame number was corresponding to the time of the electric field waveform in Fig. 2. The stepped leader kept propagating downward, lasting for about 200 μs, and then darkened at frame 11,894. At Frame 11,897, the stepped leader channel re-illuminated and had already propagated a little further than Frame 11,894. Then the stepped leader kept propagating downward for about 400 μs. After Frame 11,897, the leader channel first lighted up and then darkened, and then completely darkened at Frame 11,904.

At about Frame 11,905, the leader re-illuminated again and began to propagated bidirectionally. The upward leader propagated back to the high altitude along the preexisting channel of the decayed stepped leader, and the downward leader propagated downward along the decayed continuous current channel. The bidirectional leader, called Bidirectional leader-1 (BL-1), was unstable and decayed at Frame11913, lasting for 400 μs. Then, the second bidirectional leader (BL-2) started with the upward end traversing back to cloud along the preexisting channel of the terminated BL-1 and the other end propagating downward to the ground. The upward end of BL-2 propagated for about 350 μs and reached the cloud base at Frame 11,920. Therefore, we cannot confirm whether the leader still propagated in two directions after Frame 11,920. The downward leader kept propagating toward the ground, and finally produced RS4.

Figure 2b shows the height and 2-D estimated velocity variation of both upward positive leader (UPL) and downward negative leader (DNL) in the process of Fig. 2a. The height and velocity of leaders were calculated using two consecutive leader-visible frames. The 2-D velocity of the DNL increased slowly on the whole, and increased significantly when the leader was about to reach the ground. The height of the head of DNL showed the opposite similar trend to the velocity. The stepped leader propagated from 1.46 km above the ground to about 1.22 km. And the velocities of the stepped leader ranged from 0.20 × 10^6^ to 0.57 × 10^6^ m/s, with an average velocity of 0.40 × 10^6^ m/s.

Then the BL-1 was generated with the upward end propagating to a height of 1.41 km and the downward end propagating to a height of 1.04 km. The velocity of downward end of the BL-1 ranged from 0.40 × 10^6^ to 0.92 × 10^6^ m/s, with an average velocity of 0.67 × 10^6^ m/s. The velocity of upward end of BL-1 ranged from 0.39 × 10^6^ to 1.78 × 10^6^ m/s, with an average velocity of 0.76 × 10^6^ m/s. The velocity of upward end was found be noticeably fluctuated, and the average velocity was similar to that of the downward end. After the BL-1 decayed, the BL-2 was generated, and the velocity of DNL decreased significantly from 0.92 × 10^6^ to 0.36 × 10^6^ m/s. The velocity of downward end of BL-2 increased from 0.36 × 10^6^ to 1.30 × 10^6^ m/s, with an average velocity of 0.88 × 10^6^ m/s. And the velocity of upward end of BL-2 ranged from 1.03 × 10^6^ to 2.77 × 10^6^ m/s, with an average velocity of 1.82 × 10^6^ m/s. The speed of upward end of BL-2 was significantly faster than that of downward end of BL-2 probably due to a better channel condition of the preexisting channel of the terminated BL-1. The velocity of DNL increased up to 7.02 × 10^6^ m/s before it reached the ground and then generated a return-stroke.

Figure 2c–d shows the electromagnetic field results. The electric field was 130 m away from the rocket launcher and the magnetic field was 1.55 km away from the rocket launcher. As shown in Fig. 2a, there was almost no change in the ground electric field when the stepped leader developed. During the BL-1 development, the negative electric field began to increase slowly. With the downward negative leader approaching to the ground, the increasing trend of electric field was faster and faster before the fourth RS generated (RS process was not shown in this paper). Regarding the magnetic field, the magnetic field was featured by strong pulses during the stepped leader development and before generation of return-stroke. A significant single pulse was observed in the magnetic field when the BL-2 initiated.

**Figure 3:**
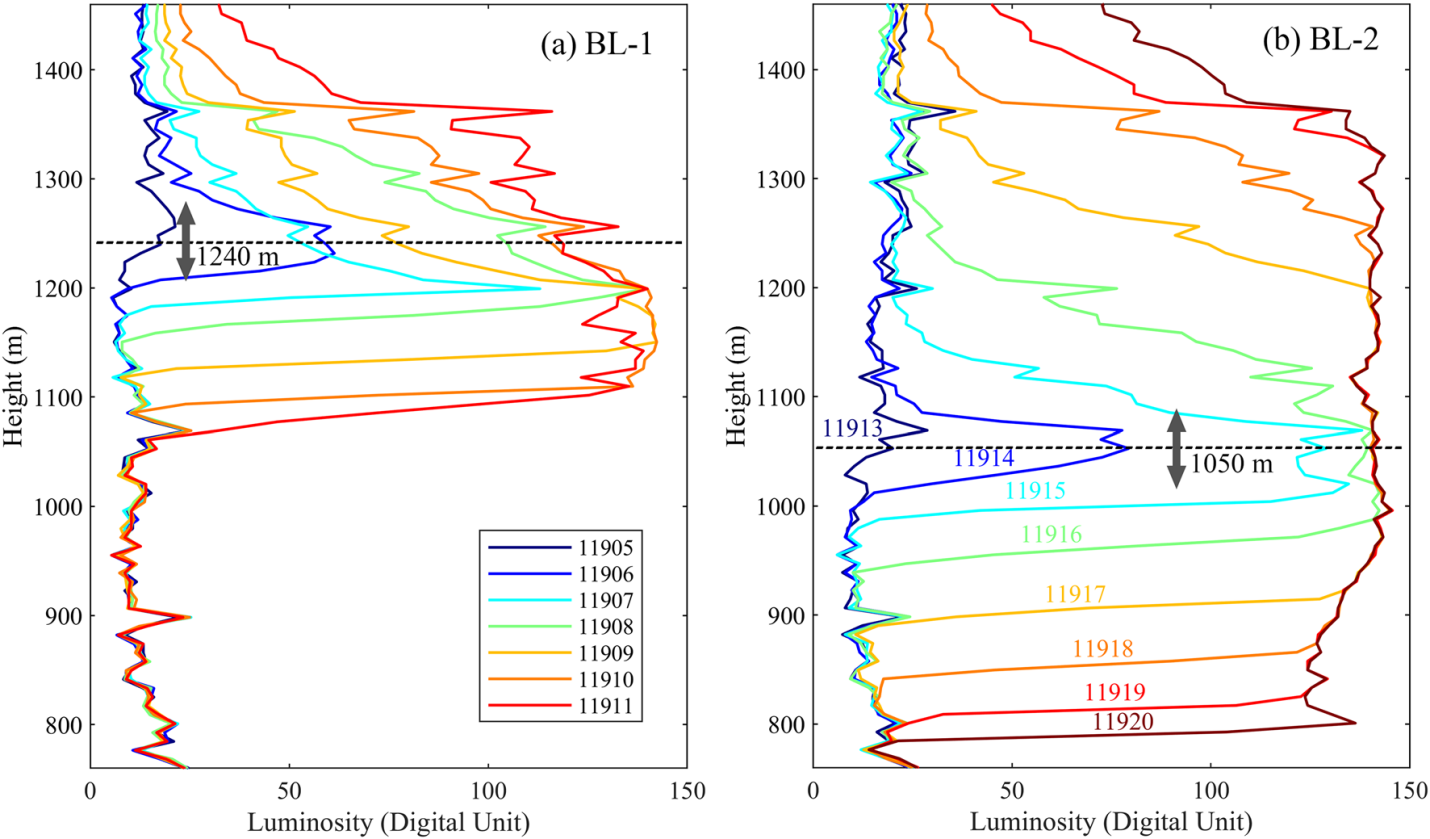
The maximum luminosity distributions of the bidirectional leader channel at different instants for the (**a**) BL-1 and the (**b**) BL-2 processes. The background noise has been removed. The legend in (**a**) and the labeled number in (**b**) corresponds to the frame number in Fig. 2a.

Figure 3 shows the maximum luminosity distributions of the bidirectional leader channel at different instants for the BL-1 and the BL-2 processes. The luminosity distribution curve of Frame 11,905 and Frame 11,913 shows the luminosity distribution before the bidirectional initiated. The luminosity of the just decayed leader channel (above 1240 m in Frame 11,905 and above 1050 m in Frame 11,913) was slightly greater than or similar to that of the residual continuous current channel (below 1240 m in Frame 11,905 and below 1050 m in Frame 11,913). The luminosity of the channel before the bidirectional leader initiated was less than 30, confirming that the leader had decayed. The leader channel that had just decayed had a higher temperature than the residual continuous current channel, resulting its slightly higher luminosity.

The luminosity distribution of the channel extending upward and downward from about 1240 m and 1050 m in Fig. 3a,b indicated the bidirectional development processes of the BL-1 and BL-2, respectively. As shown in Fig. 3a, in the BL-1 process, the upward end propagated upward as a whole, but there were also exceptional processes that do not propagated upward, such as Frame 11,908 to Frame 11,909, indicating the unsteady propagation of the BL-1. The corresponding process of the BL-2 shown in Fig. 3b was more stable with a continuously propagating upward end.

**Figure 4:**
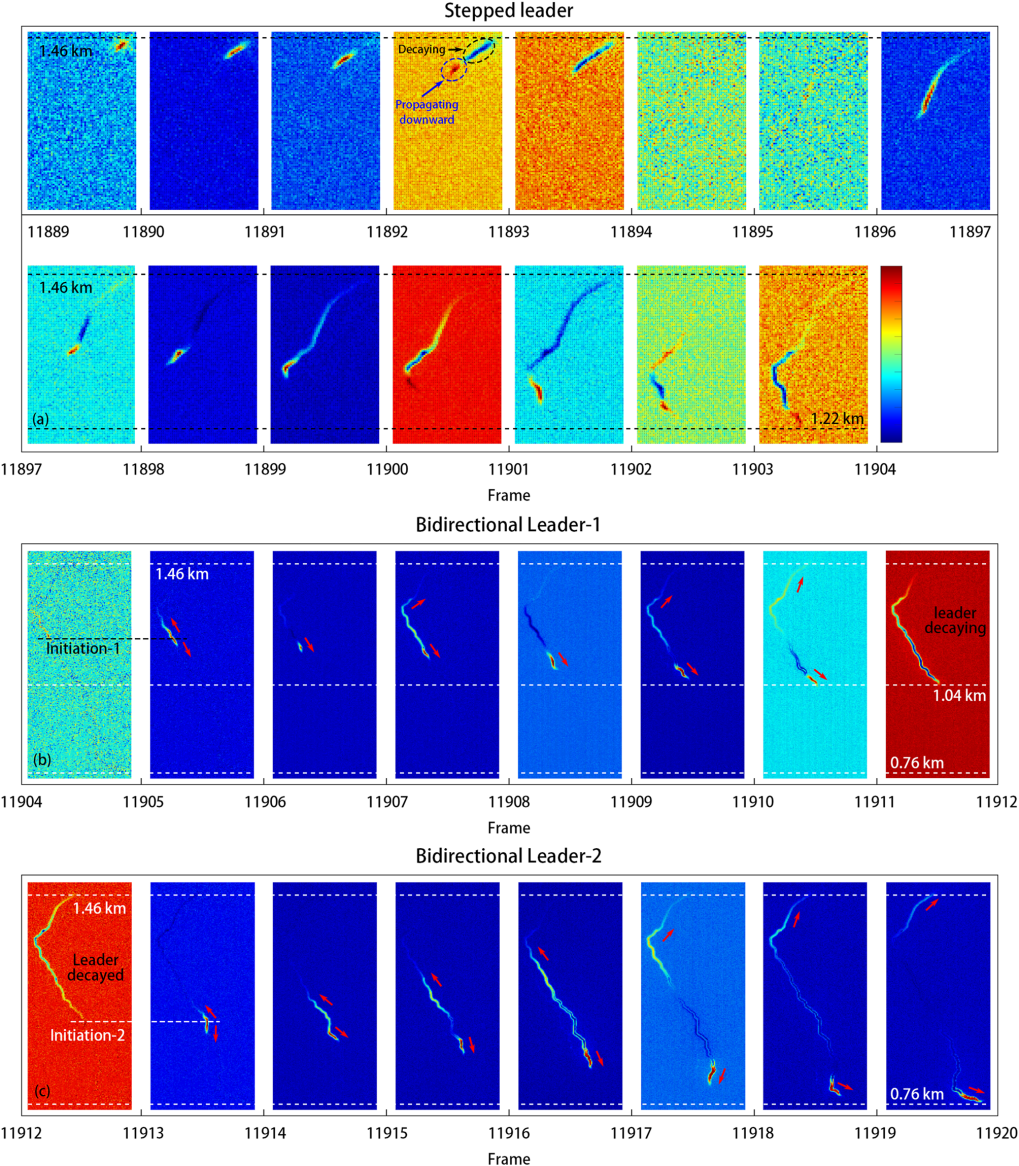
Digital differential pixel-array between two cropped consecutive frames of the stepped leader and the bidirectional leader processes. Use the light intensity of each pixel in the latter frame to minus the intensity value of the corresponding pixel in the former frame, getting the difference images.

To achieve a better view and more clearly of the change process of leader, a mathematical difference between two consecutive frames of high-speed images was proposed by using the light intensity of each pixel in the latter frame minus the intensity value of the corresponding pixel in the former frame. The methodology was named as Digital Differential Pixel-array in the present paper, and the corresponding results were shown in Figs. 3 and 4.

Figure 4 shows the development process of the stepped leader and the bidirectional leaders. As for the stepped leader, the brightness was concentrated on the head when the leader propagating downward. When the leader was going to decay, the head of the leader still propagated for a distance, and the tail channel began to decay firstly, as shown in Frame 11,893–11,892. From Frame 11,889 to Frame 11,894, the leader began to propagate and then decayed lasting for about 250 μs. After about 100 μs, at Frame 11,897–11,896, The decayed leader reillumined significantly and moved forward a certain distance, accompanied by pulses in magnetic field. Then it repeated the process of propagating forward and decaying.

As for the bidirectional leaders, the bidirectional leader initiated from the head position of the decayed downward leader. After a period of bidirectional propagation for about 50 μs, the upward end of BL-1 nearly stopped propagating from Frame 11,906 to Frame 11,907. The upward leader significantly propagated again from Frame 11,907, and then darkened from Frame 11,908 to Frame 11,909. After the propagating upward from Frame 11,909 to Frame 11,911, the whole BL-1 decayed completely. The upward leader of BL-1 was characteristic by the discontinuous upward propagation. The BL-2 initiated at Frame 11,913. The development of BL-2 was more sustained, with both ends propagating forward. When the BL-2 developed to a certain extent, the middle part of the channel tended to be stable, which might indicate that the dynamic of leader was concentrated in the head position.

## Discussion

In this paper, two successive bidirectional leader propagating in the same preexisting channel, followed by a return-stoke, were observed. The bidirectional leader reported by Montanyà et al.^5^ initiated in the virgin air below the cloud. The different polarities at both ends of the bidirectional leader lead to the asymmetric development of both ends. The negative end was characterized by a high degree of branching and bright leader tips, while the positive end was characterized by its smaller leader speed and the lack of branches. The bidirectional recoil leader usually propagated within the remnant positive leader channel as the remnant channel has a better channel condition (higher temperature and conductivity)^2,4,8,9^. The bidirectional leaders in this paper occurred in the residual continuous current channel and can be regarded as a recoil leader with the positive end retrogressing along a negative leader channel, whose polarity is contrary to the traditional recoil leader with negative leader end retrogressing along an existing positive leader channel.

Qie et al.^6^ and Jiang et al.^11^ reported high-speed video evidences of bidirectional leader development in a preexisting channel, but all of that cases had only one bidirectional leader propagating in the decayed negative channel and finally produced a return-stroke. Wu et al.^13^ reported recoil leaders producing and not producing return-strokes in a Canton-Tower upward flash, and summarized that each dart leader was preceded by one or more attempted leaders and initiated near the extremity of the positive end of the preceding attempted leader. The decayed stepped leader and the BL-1 were just like the attempted leader before the dart leader producing return-stroke.

The average velocity of stepped leader in this paper was 0.40 × 10^6^ m/s, which was consistent with the conventional velocity of the stepped leader^14–17^. The leader step pattern can be described in the following sequence^17^: (1) the streamer area forms ahead of the leader tip, (2) the streamer area and the backward leader channel increase in luminosity, (3) the streamer area and the leader channel decrease in luminosity, (4) the streamer area reilluminates ahead of the leader tip, and (5) the reilluminated streamer area develops to a new leader tip and a new streamer area is emitted forward ahead of the new leader tip. The stepped leader showed the alternating change of lightening and darkening of the whole channel, and there was no obvious bidirectional propagation, which was quite different from the later bidirectional leaders.

The brightness in the upper part of the channel persistently exists in the leading propagating process even when the leader had completely decayed. The luminosity of the just decayed leader channel was slightly greater than or similar to that of the residual continuous current channel, and the luminosity of the channel when the leader had decayed was less than 30, confirming that the leader had decayed. The higher temperature of the leader channel that had just decayed resulted its slightly higher luminosity. As no unified relationship between the channel current and luminosity could be established, the channel current cannot be inferred from the luminosity^18^. Even though the downward stepped leader terminated on the way to the ground, it showed the evidence that the lightning discharge inside the cloud is still alive and growing to force the unstable channel at lower altitude, promoting the initiation of the bidirectional leaders.

The positive leaders of bidirectional leaders in Qie et al.^6^ propagated with an average speed of 1.3 × 10^6^ m/s and 2.2 × 10^6^ m/s in the two cases, roughly twice as fast as its negative counterpart with the speed of 7.8 × 10^5^ m/s and 1.0 × 10^6^ m/s, respectively. The average 2-D partial speed of upward leader and downward leader of detected bidirectional dart leader in Jiang et al.^11^ were 6.4 × 10^6^ m/s and 2.2 × 10^6^ m/s, respectively. The bidirectional leader in Qie et al.^6^ and Jiang et al.^11^ have a common feature, that is, the positive leader propagated in the preexisting channel, and the speed of the upward positive leader is about two to three times that of the negative downward leader. The development of the decayed leader resulted in a better condition (higher channel temperature and conductivity) of the upper channel than the lower part of the channel, and there might be residual negative charge deposited in the channel, all of which promoted the upward propagation of the bidirectional leader and contributed to the higher velocity of the positive end of bidirectional leader^6,11,19^.

The positive end and negative end of the BL-1 propagated with the average velocity of 0.76 × 10^6^ m/s and 0.67 × 10^6^ m/s, respectively, which were smaller than the velocity in Qie et al.^6^ and Jiang et al.^11^ especially the positive end. Noting that the velocity of the positive and negative ends of BL-1 was similar, but the velocity of positive leader was found to be noticeably fluctuated, with the velocity ranging from 0.39 × 10^6^ to 1.78 × 10^6^ m/s. The discontinuity development of upward end of the BL-1 shown in Fig. 4 might indicate the unstable development of the BL-1, which is quite different from the bidirectional leader in Qie et al.^6^ and Jiang et al.^11^.

**Figure 5:**
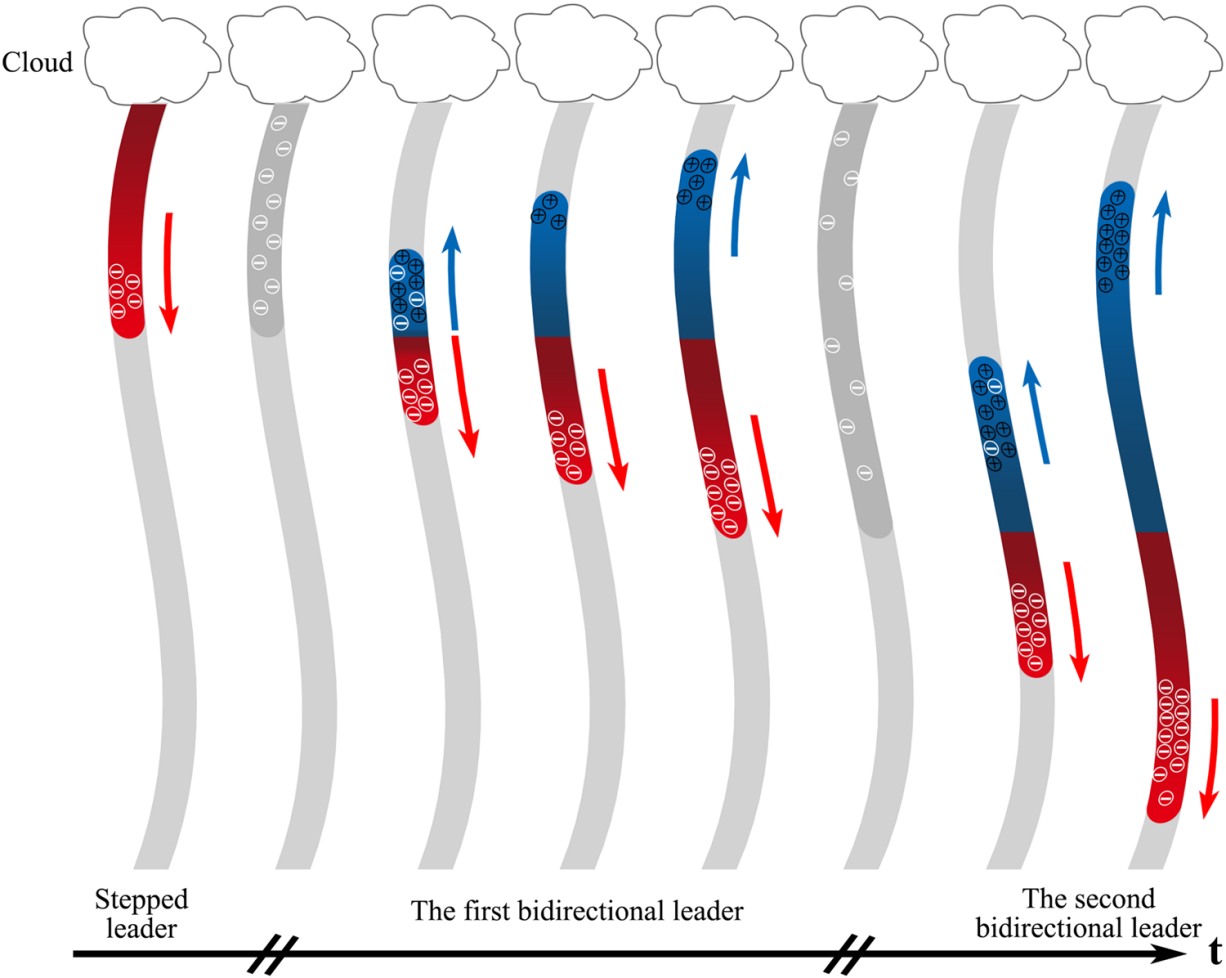
Bidirectional leader development mode. Gray part indicates residual channels. Red part indicates the negative leader, and blue part indicates the positive leader. The darker part of the leader is the plasma channel.

Figure 5 shows the bidirectional leader development mode. There was residual negative charge deposited in the channel after the preceding stepped leader decayed. Then the weak BL-1 initiated with the positive leader propagating along the upper channel. The upward channel was characterized by the deposited negative charge of the decayed steeped leader. As the amount of the charge in the head of BL-1 was relatively limited, the velocity of both ends of the BL-1 were slow. The aforementioned residual negative charge promoted the upward development of the positive leader to a certain extent. But after the positive leader propagated a certain distance, the limited positive charges were largely neutralized, resulting in insufficient of dynamic and slowdown of development of upward positive leader. On the other hand, with the development of the downward negative leader, the negative charge in the negative leader increased, and the positive charge in the positive leader increased accordingly, contributing to the reacceleration of the upward development of the positive leader. Such repetitions led to the fluctuation phenomena of the positive leader.

The positive end and negative end of the BL-2 propagated with the average velocity of 1.82 × 10^6^ m/s and 0.88 × 10^6^ m/s, respectively. Both the velocity and the velocity ratio of two end of the bidirectional leaders were consistent with the counterpart in Qie et al.^6^ The charge was supposed to be sufficient at both ends of the BL-2 to promote the propagation of the leaders. The residual negative charge only promoted the development of positive leader, while the amount of positive charge in the upward leader was almost unaffected. As a zero-net charge in the bidirectional leader^20,21^ and there was no branch in two ends of BL-2, the velocity of upward and downward leader of stable BL-2 have the same changing trend.

In conclusion, we analyzed in detail the high-speed images of two successively initiated bidirectional leaders preceded by stepped leader in triggered lightning. The BL-1 occurred following the termination of decayed stepped leader, with upward and downward average velocity of 0.76 × 10^6^ m/s and 0.67 × 10^6^ m/s, respectively. The average velocity of upward and downward leaders was similar, while the velocity of positive leader was found to be noticeably fluctuated, with the velocity ranging from 0.39 × 10^6^ to 1.78 × 10^6^ m/s. When the amount of positive charge in upward leader was limited (BL-1), the limited positive charges were largely neutralized by residual negative charge, resulting in insufficient of dynamic and slowdown of development of upward leader. The upward leader might reaccelerate when the positive charge in upward leader increases as the downward leader propagates. The BL-2 occurred following the termination of failed BL-1, with upward and downward average velocity of 1.82 × 10^6^ m/s and 0.88 × 10^6^ m/s, respectively. When the stable BL-2 initiated, the magnetic field had an obvious pulse. When there was fruitful positive charge in upward leader (BL-2), the residual negative charge only promoted the development of positive leader, while the amount of positive charge in the upward leader was almost unaffected.

## Methods

### Guangzhou triggered lightning flashed observation experiment

Artificially Triggered lightning Experiment had been carried out in the summer of 2019 in Guangzhou Field Experiment Site for Lightning Research and Testing (GFESLRT), and the flash (F201906301713) analyzed here was triggered at 17:13:13 on 30 June, 2019. The observations were performed by Engineering Research Center of Lightning Protection & Grounding Technology, Ministry of Education, China. More details about the experiment can be seen in Cai el al.^22^ and Wang et al.^23^ F201906301713 was triggered using the classical technique and recorded by a high-speed camera and fast electric field antennas at different distances.

### High-speed camera

The flash was recorded by a high-speed video camera Phantom V2512 operated at a framing rate of 20,000 frames per second (time resolution is 50 μs) with exposure time of 49 μs. The spatial resolution was 640 × 608 (horizontal × vertical) pixels. Each pixel had a length of 28 μm and lens focal length of 16 mm, with a distance from camera to lightning launcher of 1.55 km, providing a spatial resolution of 2.71 m per pixel.

### Electric fields and current measurement

The current of the flash was measured by a coaxial shunt with a resistance of 1 mΩ at the bottom of the triggering facility. The current signal was transmitted to the control room 130 m away from the rocket launcher via a fiber transmission system. The electric field was measured by the fast antenna 3.6 m above the ground. The current signal and the electromagnetic field signal at 130 m were digitized at a 50 MS/s sampling and a recording length of 2 s. And the electromagnetic measurements were also installed on the house roof 1.55 km away from the rocket launcher, with a sampling rate of 5 MS/s. Measurements were synchronized using GPS timing.

### The methodology of digital differential pixel-array

To achieve a better view and more clearly of the change process of leader, a mathematical difference between two consecutive frames of high-speed images was proposed by using the light intensity of each pixel in the latter frame minus the intensity value of the corresponding pixel in the former frame. The methodology was named as Digital Differential Pixel-array (DDP) in the present paper. The color range of each image is from the minimum value to the maximum value of the differential light intensity. The color of these images represents the relative and differential light intensity. The color of the background part corresponded to the zero value of the differential light intensity in general, indicating that the luminosity remains unchanged. According to the color bar in Fig. 4, the color above the background color indicated an increase in light intensity, and the color below the background color indicated a decrease in light intensity.

## Data Availability

The datasets used and analyzed during the current study available from the corresponding author on reasonable request.

## References

[1] Kasemir HW (1960). A contribution to the electrostatic theory of a lightning discharge. J. Geophys. Res..

[2] Warner TA, Cummins KL, Orville RE (2012). Upward lighting observations from towers in Rapid City, South Dakota and comparison with National Lightning Detection Network data, 2004–2010. J. Geophys. Res..

[3] Rakov VA (1998). New insights into lightning processes gained from triggered-lightning experiments in Florida and Alabama. J. Geophys. Res..

[4] Kotovsky D, Uman MA, Wilkes RA, Jordan DM (2019). High-speed video and lightning mapping array observations of in-cloud lightning leaders and an M component to ground. J. Geophys. Res. Atmos..

[5] Montanyà J, van der Velde O, Williams ER (2015). The start of lightning: Evidence of bidirectional lightning initiation. Sci. Rep..

[6] Qie X, Pu Y, Jiang R, Sun Z, Liu M, Zhang H, Li X, Lu G, Tian Y (2017). Bidirectional leader development in a preexisting channel as observed in rocket-triggered lightning flashes. J. Geophys. Res. Atmos..

[7] Chen ML, Watanabe T, Takagi N, Du YP, Wang DH, Liu XS (2003). Simultaneous observations of optical and electrical signals in altitude-triggered negative lightning flashes. J. Geophys. Res..

[8] Mazur V, Ruhnke LH (2011). Physical processes during development of upward leaders from tall structures. J. Electrostat..

[9] Mazur V, Ruhnke LH, Warner TA, Orville RE (2013). Recoil leader formation and development. J. Electrostat..

[10] Tran MD, Rakov VA (2016). Initiation and propagation of a cloud-to-ground lightning observed with high-speed video camera. Sci. Rep..

[11] Jiang R, Wu Z, Qie X, Wang D, Liu M (2014). High-speed video evidence of a dart leader with bidirectional development. Geophys. Res. Lett..

[12] Rakov VA, Uman MA (2003). Lightning: Physics and Effects.

[13] Wu B, Lyu W, Qi Q, Ma Y, Chen L, Jiang R (2019). High-speed video observations of recoil leaders producing and not producing return strokes in a Canton-Tower upward flash. Geophys. Res. Lett..

[14] Wang F, Wang J, Cai L, Su R, Ding W, Xu Z (2021). Leader-chasing behavior in negative artificial triggered lightning flashes. Sci. Rep..

[15] Schonland, B. F. J. The lightning discharge. In *Handbuch der Physik*, vol. 22, 576–628 (Springer-Verlag, 1956).

[16] Shao XM, Krehbiel PR, Thomas RJ, Rison W (1995). Radio interferometric observations of cloud-to-ground lightning phenomena in Florida. J. Geophys. Res..

[17] Huang H, Wang D, Wu T, Takagi N (2018). Formation features of steps and branches of an upward negative leader. J. Geophys. Res. Atmos..

[18] Qie X, Jiang R, Wang C, Yang J, Wang J, Liu D (2011). Simultaneously measured current, luminosity, and electric field pulses in a rocket-triggered lightning flash. J. Geophys. Res..

[19] van der Velde OA, Montanya J (2013). Asymmetries in bidirectional leader development of lightning flashes. J. Geophys. Res. Atmos..

[20] Vargas M, Torres H (2008). On the development of a lightning leader model for tortuous or branched channels—Part I: Model description. J. Electrostat..

[21] Mansell ER, MacGorman DR, Ziegler CL, Straka JM (2002). Simulated three-dimensional branched lightning in a numerical thunderstorm model. J. Geophys. Res..

[22] Cai L, Li J, Wang J, Su R, Wang F, Li Q (2021). Optical progressing and electric field change characteristics of altitude—Triggered lightning flash with different development paths. J. Geophys. Res. Atmos..

[23] Wang J, Wang S, Cai L, Lu D, Li Q, Zhou M, Fan Y (2020). Observation of induced voltage at the terminal of 10 kV distribution line by nearby triggered lightning. IEEE Trans. Power Deliv..

